# Subjective benefits from wearing self-fitting over-the-counter hearing aids in the real world

**DOI:** 10.3389/fnins.2024.1373729

**Published:** 2024-04-18

**Authors:** Tong Sheng, Lauren Pasquesi, Jennifer Gilligan, Xing-Jie Chen, Jayaganesh Swaminathan

**Affiliations:** Eargo, Inc., San Jose, CA, United States

**Keywords:** hearing loss, over-the-counter hearing aids, patient satisfaction, quality of life, SADL

## Abstract

**Introduction:**

In 2022, the US Food and Drug Administration enacted final regulations to establish the category of over-the-counter (OTC) hearing aids aimed at reducing barriers to access hearing health care for individuals with self-perceived mild to moderate hearing loss. However, given the infancy of this device category, the effectiveness of OTC hearing aids in real-world environments is not yet well understood.

**Methods and results:**

To gain insights into the perceived benefit of self-fitting OTC hearing aids, a two-pronged investigation was conducted. In the primary investigation, 255 active users of a self-fitting OTC hearing aid were surveyed on their perceived benefit using an abridged form of the Satisfaction with Amplification in Daily Living (SADL) scale. The mean global (4.9) and subscale scores (Positive Effect (PE): 4.3; Negative Features (NF): 4.3; Personal Image (PI): 6.1) were within the range of those previously reported for users of prescription hearing aids. In the secondary investigation, 29 individuals with self-reported hearing impairment but no prior experience with the investigational self-fitting OTC hearing aids used the devices and reported their perceived benefit and satisfaction following short-term usage. For this prospective group, the global SADL (5.4) and subscale scores (PE: 4.8; NF: 4.9; PI: 6.5) following a minimum of 10 weeks of real-world use were also within the range of those previously reported for traditional hearing aid users. In addition, this prospective group was also asked quality of life questions which assessed psychological benefits of hearing aid use. Responses to these items suggest hearing aid related improvements in several areas spanning emotional health, relationships at home and at work, social life, participation in group activities, confidence and feelings about one’s self, ability to communicate effectively, and romance.

**Discussion:**

Converging data from these investigations suggest that self-fitting OTC hearing aids can potentially provide their intended users with a level of subjective benefit comparable to what prescription hearing aid users might experience.

## 1 Introduction

According to the National Institute on Deafness and Other Communication Disorders (NIDCD), approximately 28.8 million adults in the United States could benefit from the use of hearing aids (NIDCD, 2021). Hearing loss is a prevalent condition with approximately 40 million adults (15%) in the United States reporting having at least a little trouble with hearing (Pleis and Lethbridge-Cejku, 2007). Hearing loss is disproportionately overrepresented among older adults, with nearly 25% of American adults aged 45 years and older reporting trouble hearing (Pleis and Lethbridge-Cejku, 2007), and its prevalence doubling with each additional decade of life (Lin et al., 2011).

In addition to affecting one’s ability to communicate, hearing loss has also been associated with adverse physical and mental health outcomes. Among older adults over 70 years of age, those with hearing loss were also more likely to have a history of cardiovascular disease and stroke, resulting in an increased mortality risk (Contrera et al., 2015). Hearing loss is also associated with worse depressive symptoms (West et al., 2023), greater prevalence of dementia (Huang et al., 2023), higher rates of difficulties in activities of daily living (Dalton et al., 2003; Choi et al., 2016), and poorer quality of life (QoL), especially pertaining to social and emotional relationships (Ciorba et al., 2012).

While associations between hearing loss and adverse health outcomes are correlational, hearing loss is considered a modifiable risk factor, and its rehabilitation may have some potential to lessen the deterioration of health and quality of life. Early screening and adoption of hearing aid use can play a role in maintaining a positive quality of life (Brodie et al., 2018), and hearing aid use has also been associated with reduced anxiety and depression symptoms, improved QoL indicators, and reduced hearing-related social and emotional impediments (Ciorba et al., 2012). Among older adults with moderate to severe hearing loss, hearing aid use has been associated with lower prevalence of dementia (Huang et al., 2023), and a recent intervention study showed that older adults who were at greater risk of cognitive decline showed less cognitive decline following hearing aid use than those who did not use hearing aids (Lin et al., 2023).

Although the evidence for the rehabilitative benefits of hearing aids continues to accumulate, widespread hearing aid adoption has been stymied by factors such as lack of awareness and motivation (Angara et al., 2021; Zheng et al., 2023), as well as difficulty accessing hearing healthcare, all of which may contribute to the delayed diagnosis and treatment of hearing loss. Furthermore, while hearing aids are viewed as a relatively cost-effective rehabilitation tool, disparate insurance reimbursement policies and potentially high out-of-pocket costs can present a financial barrier to entry (Jilla et al., 2023). These factors can lead to individuals with hearing loss to not seek hearing healthcare altogether or only begin to use hearing aids after their condition has already worsened significantly.

Although the NIDCD reports the need for hearing aid adoption is high, usage continues to be low with 30% utilization for hearing aid candidates aged 70 and above and 16% utilization for candidates aged 20 to 69 (NIDCD, 2021). In recognition of this, the United States Food and Drug Administration (FDA) established a new category of over-the-counter (OTC) hearing aids for adults with self-perceived mild to moderate hearing impairment. By creating the OTC hearing aid category, the FDA hopes to reduce barriers to access and readily unlock the benefits associated with hearing aid use for those with hearing loss (FDA, 2023).

The OTC category of hearing aids aims to promote hearing aid adoption and use through two main objectives: 1) by establishing a category of devices that is accessible independent of the involvement of a hearing healthcare professional, and 2) by ensuring that the devices can be controlled (i.e., adjusted) directly by the end user (21CFR800.30, n.d.). By definition, OTC hearing aids are air-conduction hearing aids that do not require a hearing healthcare professional to procure or prescribe, and do not require implantation or other surgical means to fit to a user’s ears. Such devices must also have user controls that enable the end user to adjust the devices based on their hearing needs.

To mitigate the risks associated with making such medical devices available to a potentially broad user base, device manufacturers must satisfy a range of controls in order for a product to meet the requirements of an OTC hearing aid (21CFR800.30, n.d.). These controls include software labeling, device output limits (i.e., maximum acoustic output limits), electroacoustic performance (e.g., distortion, latency, frequency response), and design requirements (e.g., maximum insertion depth, use of atraumatic materials, user controls).

There are broadly two kinds of OTC hearing aids: those with preset amplification levels, and those that are self-fitting. Self-fitting OTC hearing aids, which can be customized based on an individual’s hearing loss, require FDA 510(k) clearance, including submission of clinical data, to validate the effectiveness of the self-fitting strategy. Self-fitting OTC hearing aids aim to be easily-accessible and user-friendly, as they can be obtained and fit without the involvement of a hearing healthcare professional. Removing the need to be seen in-person by a hearing care professional may encourage more people with hearing loss to use hearing aids, and at earlier stages of their hearing loss progression, by providing direct access to OTC devices.

While self-fitting OTC hearing aids intend to be more accessible, whether these devices will be perceived as beneficial in isolation and/or compared to prescription hearing aids fit by an audiologist following real world device wear by its users is yet to be seen. Clinically validated questionnaires have been developed to investigate the satisfaction of hearing aid users with real world device wear. Among the most known and used is the Satisfaction with Amplification in Daily Life questionnaire (SADL; Cox and Alexander, 1999). The SADL was developed to ascertain an overall sense of a user’s satisfaction with hearing aids, as well as satisfaction in more specific areas related to hearing aid procurement and use (e.g., positive effect, service and cost, negative features, and personal image). Thus, the SADL aimed to quantify the degree of satisfaction with the use of a hearing aid, its perceived benefit, and allow for the identification of adverse aspects of adaptation of hearing aids.

While the real-world benefits and satisfaction with prescription hearing aids (fit by an audiologist following clinical best practice methods) have been extensively studied using surveys such as SADL, perceived benefit from the use of OTC hearing aids has been minimally studied. In the current study, we seek to gain insight into the extent to which users of a commercially-available, FDA-cleared self-fitting OTC hearing aid system report satisfaction and a sense of perceived benefit following real-world device use. To gain insights into the perceived benefit of OTC hearing aids, we conducted 1) a retrospective satisfaction survey study involving current users of an FDA-cleared self-fitting OTC hearing aids, and 2) a prospective cohort study with individuals who fit the description of the intended users of OTC hearing aids who had not previously used the investigational self-fitting OTC hearing aids.

The retrospective satisfaction survey would provide a better understanding of the impact of self-fitting hearing aids after its users acclimatized to and integrated the devices into their everyday lives, whereas the prospective cohort study would provide insight into the onboarding journey and usage experience of those who are new to the investigational self-fitting hearing aids.

## 2 Methods

In the context of these investigations, the study devices were commercially available Eargo hearing (www.eargo.com; San Jose, CA) aids that have been cleared by the FDA to be marketed as a Class II self-fitting air-conduction hearing aid (K221698, n.d.) and meet the controls set forth by the OTC rule (21CFR800.30, n.d.). The Eargo self-fitting OTC hearing aid system consists of a pair of completely-in-the-canal (CIC) style hearing aids (left and right), a charging case, and a companion mobile app. The Eargo self-fitting hearing aid uses a proprietary method that requires the user to complete a self-guided hearing assessment using the mobile app while wearing the hearing aids. The hearing aids act as the transducer, emitting tonal stimuli of varying levels at different audiometric frequencies. The measured hearing thresholds are then used as the basis for fitting the appropriate gain settings for the user. Once fit, the user can make additional adjustments (e.g., volume, bass/treble) to the left, right, or both hearing aids using the mobile app to achieve a desired fitting. Eargo’s self-fitting hearing aids have been rigorously clinically validated and has been shown to provide adults with mild to moderate hearing loss with functional performance that is non-inferior to that provided by a professional hearing aid fitting (Hu et al., 2022; Urbanski et al., 2022; K221698, n.d.).

### 2.1 Retrospective self-fitting OTC hearing aid use satisfaction

To gain an understanding of user satisfaction and perceived benefit associated with the use of Eargo self-fitting OTC hearing aids, we leveraged the Eargo user base to identify individuals who had purchased an Eargo self-fitting OTC hearing aid and who had completed the product’s self-fitting feature using its companion mobile application. In addition, we limited the query to identify only those who have purchased their devices at least 90 days prior to executing the query to constrain the sample to those who have had a chance to acclimatize to the hearing aids.

A random sample of subjects among those meeting the above criteria were invited to participate in a web-based survey about their experience using Eargo self-fitting OTC devices. Participation in the survey study was completely voluntary, and those who consented to participate in the survey received compensation in the form of a $25 Amazon gift card. The survey consisted of device usability and satisfaction questions, including questions from an abridged form of the Satisfaction with Amplification in Daily Living (SADL) questionnaire (Cox and Alexander, 1999).

The SADL scale is a 15-item questionnaire that assesses satisfaction with the use of hearing aids. With the SADL, hearing aid satisfaction can be interpreted using a global score as well as four subscale scores. The global and subscale satisfaction scores are interpreted on a 7-point scale, with 1 corresponding to least satisfaction (“Not At All”) and 7 corresponding to the greatest satisfaction (“Tremendously”). There are four items that are phrased in the negative, and therefore, reverse scored. The scoring of the global and individual subscales is otherwise straight-forward, with the global score calculated as the mean score of all items completed by the participant, and individual subscale scores calculated as the mean score of all items completed by the participant within each subscale. The derived subscale and global satisfaction scores are interpreted with higher scores corresponding to higher satisfaction.

The individual subscales are: Positive Effect (PE): assessing functional benefit and satisfaction with overall hearing aid sound quality and use; Service & Cost (SC): assessing the fitting professional, product cost, and reliability/maintenance of hearing aids; Negative Features (NF): assessing the satisfaction with acoustic performance and feedback in specific challenging conditions; and Personal Image (PI): assessing the satisfaction with the hearing aids’ *in-situ* physical appearance and perceived stigma when wearing hearing aids.

The SADL inventory was originally developed to evaluate satisfaction with prescription hearing aids well before the OTC category of hearing aids was established. Therefore, items related to SC may not accurately assess user sentiment in the context of OTC hearing aids, nor offer a meaningful interpretation of its score compared to the published norms for prescription hearing aid use. As such, questions related to SC were not included in the abridged SADL questionnaire administered to the users of Eargo self-fitting OTC hearing aids. The scoring instructions permit the omission of individual items with respect to subscale and global scores. However, as the omission of individual items impacts the calculation of the global SADL score, this metric should be interpreted to exclude the service and cost aspects of obtaining and using hearing aids and with caution while comparing with published normative data. However, the individual subscale satisfaction scores related to PE, NF, and PI do offer a more direct comparison with published norms for prescription hearing aids.

### 2.2 Prospective cohort: self-fitting OTC hearing aid use satisfaction

To gain an understanding of user satisfaction and perceived benefit associated with the first-time use of Eargo self-fitting OTC hearing aids among OTC hearing aid candidates, we recruited individuals who met the description for OTC hearing aid intended users, and who had no prior experience with Eargo’s self-fitting OTC hearing aid products, to participate in a prospective cohort study. Potential candidates were recruited for screening via local advertising, word of mouth, and a customer database search. Intended users of OTC hearing aids were defined by the FDA as adults with self-reported mild-to-moderate hearing impairment, and this included individuals who have trouble hearing speech in noisy places, find it difficult to follow speech in groups, have trouble hearing on the phone, become tired when listening, and need to turn up the volume on the TV or radio to a level where other people complain it’s too loud.

Participants who met the criteria described above and who consented to participating in the study were provisioned with retail-equivalent Eargo self-fitting OTC hearing aids (including all product package labeling and instructions for use that would accompany the system as if it were purchased commercially), along with a retail-equivalent investigational companion mobile app.

To approximate the journey of a would-be retail client of Eargo self-fitting OTC hearing aids, we asked participants to wear the devices to the extent that they found appropriate or desirable, and provided no further instructions apart from requesting that they perform the app-based self-fitting procedure. This was to ensure that participants experienced the self-fitting process and that they would be testing and providing feedback on a self-fit hearing aid system. Otherwise, participants were expected to navigate their own hearing aid onboarding journey by using their devices as often or occasionally as they wished, and to review the included instructional materials for device troubleshooting. Participants were allowed to contact study staff if they had any questions, and research staff provided a scope and extent of support that mirrored those available to retail clients.

While all enrolled participants had to meet the criterion of not having prior experience with Eargo self-fitting OTC hearing aids, they were not excluded if they had previously tried or used other hearing devices.

Participants were given at least 10 56 weeks to become familiar with the study devices and to use the devices as much or as little as they felt appropriate in their everyday lives. At the conclusion of the study, all participants were administered a web-based survey on their experiences and satisfaction with using the study device and provided compensation in the form of a $75 Amazon gift card for participating in the study. As the study enrollment occurred on a rolling basis, and the final survey administration occurred at a fixed time point, several participants spent more than the minimum 10 weeks testing the study devices.

The survey consisted of device usability and satisfaction items, including the same abridged SADL questionnaire described above for the retrospective study. Overall satisfaction (global SADL score) and satisfaction in PE, NF, and PI were assessed. In addition, subjects were also asked quality of life (QoL) questions adapted from the MarkeTrak VIII survey (Kochkin, 2011). These questions assessed whether hearing aid users endorsed improvements across various QoL domains – emotional health, mental ability (memory), physical health, relationships at home, relationships at work, social life, feelings about oneself, ability to participate in group activities, sense of independence, sense of safety, confidence in oneself, sense of humor, romance in one’s life, and overall ability to communicate more effectively – that participants believed to be attributable to hearing aid use. These questions were administered by asking respondents to “rate the changes you have experienced in the following areas, that you believe are due to your hearing aids” and each scored on a 4-point scale from 1 = “Worse” to 4 = “A lot better.”

## 3 Results

### 3.1 Retrospective self-fitting OTC hearing aid use satisfaction

We identified a random sample of 393 Eargo self-fitting hearing aid subjects, and among these, 255 subjects met the inclusion criterion of having completed self-fitting using their hearing aids and the mobile app, and completed the abridged SADL questionnaire (see Table 1 for sample characteristics). Most of the respondents were experienced everyday users of the devices; nearly two-thirds had used their devices for more than 6 months (65.1%), over three-quarters reported using their devices at least 3 or more days per week (77.6%; Table 1), and nearly half reported using their hearing aids for at least 8 h a day (45.5%). The sample was evenly split with respect to the severity of self-reported hearing impairment (49% mild; 46.3% moderate).

**Table 1:** Retrospective and prospective sample characteristics.

	Retrospective sample characteristics	Prospective sample characteristics
Sample size:	*N* = 255	*N* = 29
Age:	69 years (median)	70 years (median)
	62–74.5 years (interquartile range)	64–77 years (interquartile range)
Gender:	79.2% male	72.4% male
	20.4% female	27.6% female
	0.4% other/prefer not to say	0% other/prefer not to say
Self-reported hearing difficulty	Mild: 49%	Mild: 37.9%
	Moderate: 46.3%	Moderate: 62.1%
	Severe: 4.7%	Severe: 0%
Self-reported device usage (weekly)	1–2 days/week: 22.4%	1–2 days/week: 14.3%
	3–5 days/week: 28.6%	3–5 days/week: 50%
	6–7 days/week: 49%	6–7 days/week: 35.7%
Self-reported device usage (daily)	<4 h/day: 22%	<4 h/day: 14.3%
	4–8 h/day: 32.5%	4–8 h/day: 28.6%
	8+ hours/day: 45.5%	8+ hours/day: 57.1%
Eargo self-fitting hearing aid use history	3–6 months: 34.9%	<1 month: 6.9%
	6–12 months: 48.2%	1–2 months: 51.7%
	>12 months: 16.9%	>2 months: 37.9%
Self-reported lifetime hearing aid use history	<1 year: 46.3%	<1 year: 28%
	1–10 years: 49%	1–10 years: 69%
	>10 years: 4.7%	>10 years: 3%

The global, as well as individual subscales satisfaction scores derived from the abridged SADL questionnaire, were mostly positive (Table 2). The mean modified global satisfaction (absent SC items) score of 4.9 was comparable to published satisfaction scores for traditional hearing aids obtained through private practice and fit by an audiologist following clinical best practice methods (study mean = 4.9 vs. norm mean of 4.9; T = 0.25, *p* = 0.80). For the subscale scores, the Positive Effect was slightly poorer than published norms (study mean = 4.3 vs. norm mean of 4.9), while Negative Features (study mean = 4.3 vs. norm mean of 3.6), and Personal Image (study mean = 6.1 vs. norm mean of 5.6) subscale scores were better than published norms. While the differences in subscale scores were statistically different from published norms (all Ps < 0.05), the interquartile range for each subscale overlapped with the previously reported ranges (i.e., 20th-80th percentile ranges) for users of prescription hearing aids (Cox and Alexander, 1999). A post-hoc power calculation indicated that the study had sufficient power (100%) to detect a satisfaction score difference of 0.5 (with standard deviation of 1.0) at alpha = 0.05 with the 255 respondents.

**Table 2:** Retrospective and prospective cohort hearing aid satisfaction: SADL global and subscale scores.

SADL	Retrospective sample satisfaction results *N* = 255		Prospective sample satisfaction results *N* = 29		Published norms from Cox and Alexander (1999)
Global	MEAN^a^: 4.9	T = 0.25	MEAN^a^: 5.4	**T = 4.08**	MEAN: 4.9
	SD: 0.9	*p* = 0.80	SD: 0.7	**P = 0.0003**	20th–80th range: 4.2–5.9
	IQR: 4.2–5.6		IQR: 4.8–6.1		
Positive effect	MEAN: 4.3	**T = 7.21**	MEAN: 4.8	T = 0.33	MEAN: 4.9
	SD: 1.3	**p < 0.0001**	SD: 1.2	*p* = 0.75	20th–80th range: 3.8–6.1
	IQR: 3.3–5.3		IQR: 3.8–5.8		
Negative features	MEAN: 4.3	**T = 8.65**	MEAN: 4.9	**T = 5.45**	MEAN: 3.6
	SD: 1.3	**P < 0.0001**	SD: 1.3	**P < 0.0001**	20th–80th range: 2.3–5.0
	IQR: 3.3–5.3		IQR: 4.0–6.0		
Personal image	MEAN: 6.1	**T = 11.77**	MEAN: 6.5	**T = 7.97**	MEAN: 5.6
	SD: 0.7	**P < 0.0001**	SD: 0.6	**P < 0.0001**	20th–80th range: 5.0–6.7
	IQR: 5.7–6.7		IQR: 6.2–6.8		

a The global satisfaction score derived from the abridged SADL questionnaire in the present study omits questions related to Service and Cost. Thus, its interpretation should be considered to not pertain to the Service and Cost aspects of obtaining and using hearing aids. Bold indicates *p* < 0.05 (two-tailed).

### 3.2 Prospective cohort: self-fitting OTC hearing aid use satisfaction

Thirty-three adults were enrolled into the prospective cohort study and twenty-nine subjects provided responses on the final survey. For this cohort, 37.9% self-reported having mild hearing impairment, while 62.1% self-reported having moderate hearing impairment (Table 1). The vast majority reported regularly using the study devices for at least 1 month (89.6%), with 37.9% reporting using the study devices for at least 2 months (Table 1). With respect to device usage, 85.7% reported using the devices at least 3 or more days per week, and 57.1% reporting using the devices for 8 or more hours per day.

Among this cohort of OTC hearing candidates who were new to using Eargo self-fitting hearing aids, the levels of self-reported satisfaction following this short-term device trial were within the expected range of satisfaction scores for prescription hearing aids (Table 2). Notably, the modified global satisfaction (absent SC items) following short-term wear was significantly higher than the global satisfaction score reported for prescription hearing aid users (study mean = 5.4 vs. norm mean of 4.9; T = 4.08, *p* = 0.0003). Satisfaction scores in the Negative Features (mean = 4.9) and Personal Image (mean = 6.5) subscales were significantly higher than published norms for prescription hearing aids (all *p*s < 0.05), although the interquartile range of individual SADL subscales overlapped with the ranges (i.e., 20th-80th percentile ranges) published for prescription hearing aids (Cox and Alexander, 1999).

With respect to self-reported QoL improvements attributable to the short-term use of Eargo self-fitting hearing aids, there was near-unanimous endorsement of stability or improvement in all domains assessed (at least 96% of respondents reported “same” or “better” on all 14 questions). In the following QoL domains, more than half of the responding sample reported improvements stemming from wearing self-fitting hearing aids: emotional health (54.5%), relationships at home (64%), relationships at work (61.1%), social life (65.4%), feeling about oneself (60.9%), ability to participate in group activities (60%), confidence in oneself (54.2%), romance (100%), and overall ability to communicate more effectively (69.2%; Table 3). A post-hoc power calculation indicated that while the initial sample of 33 participants demonstrated sufficient power (>80%) to detect a satisfaction score difference of 0.5 (standard deviation of 1.0) at alpha = 0.05, the sample of 29 respondents yielded a power of 76.8%.

**Table 3:** Prospective cohort quality of life changes attributed to hearing aid use.

Quality of life domain (number of respondents)	Worse	Same	Better
Romance in my life (*n* = 22)	0%	0%	100%
Overall ability to communicate more effectively in most situations (*n* = 25)	0%	26.9%	69.2%
Social life (*n* = 26)	3.8%	30.8%	65.4%
Relationships at home (*n* = 25)	0%	36%	64%
Relationships at work (*n* = 18)	5.6%	33.3%	61.1%
Feelings about yourself (*n* = 23)	0%	39.1%	60.9%
Ability to participate in group activities (*n* = 25)	4%	36%	60%
Emotional health (*n* = 22)	0%	45.5%	54.5%
Confidence in yourself (*n* = 25)	0%	45.8%	54.2%
Mental ability (memory) (*n* = 21)	0%	52.4%	47.6%
Sense of independence (*n* = 23)	0%	60.9%	39.1%
Sense of safety (*n* = 23)	0%	60.9%	39.1%
Sense of humor (*n* = 24)	0%	75%	25%
Physical health (*n* = 21)	0%	81%	19%

## 4 Discussion

This study assessed subjective benefits and satisfaction with real-world device wear using a clinically validated questionnaire (SADL) for an FDA-cleared self-fitting OTC hearing-aid system (Eargo) in adults with self-perceived hearing difficulties. Two cohorts were recruited for this study: 1) A retrospective cohort with longer acclimatization and integration of self-fitting OTC hearing-aids into their everyday lives; and 2) A prospective cohort who were new to the investigational self-fitting hearing aids. While comparing between cohorts, the mean global and subscale satisfaction scores were better for the prospective cohort (vs. scores from retrospective cohort). While it may be tempting to interpret these differences as a stabilization of perceived benefit over time (for example, initial excitement may be driving higher satisfaction in the prospective cohort), any comparison and interpretation of SADL scores between the two cohorts should be done with caution due to differences in sample size and characteristics.

However, comparisons between SADL scores from our retrospective and prospective cohorts and those reported in the literature for individuals wearing prescription hearing aids (fit by an audiologist following clinical best practice methods) can offer interesting insights. The mean modified global SADL satisfaction score (absent SC items) from the retrospective group was statistically similar to those reported in the literature for adults fit with prescription hearing aids (Cox and Alexander, 1999, 2001; Shi et al., 2007; Kozlowski et al., 2017), while the global SADL score observed in our prospective cohort was slightly elevated. This suggests that users of our investigational self-fitting OTC hearing aids who have had an opportunity to acclimatize to the devices experience a comparable level of overall satisfaction and benefit as those who have been fit with prescription hearing aids, whereas the brand new investigational device users in our prospective cohort could have exhibited some initial product excitement that may or may not temper over time.

With respect to the SADL subscale scores, the observed mean PE score in the retrospective cohort was slightly poorer than published norms, while the observed mean PE score in the prospective cohort was comparable to published norms. The PE subscale consists of items related to a device’s functional performance and whether use of the device is worthwhile to the user. It is possible that hearing aid candidates who sought treatment through the traditional channel may be better aligned with respect to their expectations when embarking on their hearing aid journey. The higher satisfaction in our prospective cohort relative to the retrospective cohort could be due to the fact that these users were provisioned investigational devices as part of a product usability study, and did not obtain them through a retail or prescription channel.

With respect to the NF and PI subscales, converging evidence from both the retrospective and prospective cohorts indicate that the observed NF and PI subscale scores following use of the Eargo self-fitting OTC hearing aids were slightly better than the published norms for prescription hearing aid users. The favorable NF scores in both of our cohorts indicate that users felt the investigational self-fitting OTC hearing aids had good acoustic performance (i.e., adequate gain with acceptable amount of feedback). For the positive PI scores in both cohorts, a reasonable explanation could be that the CIC form factor of the Eargo self-fitting OTC devices were less visually obvious than other form factors when worn *in-situ*, which may in turn alleviate some of our users’ concerns related to social stigma around hearing aid wear (Pasquesi et al., 2023).

However, although we have observed some slight differences in the mean scores across the global and subscale scores between our two cohorts and the published norms for prescription hearing aid users, the distributions (i.e., interquartile ranges) of all of our observed scores were largely comparable to the distributions (i.e., published 20th-80th percentile ranges, see Table 2) reported for prescription hearing aids. This, along with a few other study-specific details that may contribute to the interpretation of our data, encourage us to refrain from making absolute statements about satisfaction relative to prescription hearing aids.

For example, only individuals who have ordered a self-fitting OTC hearing aid system at least 90 days prior to the survey administration were eligible to participate in our retrospective study. This meant that any customers who have tried but returned their devices within the initial trial period were not included in the sample. While we did not otherwise exclude any potential participant based on complaints, return requests, customer support cases, or any other obvious indicators of device dissatisfaction, it is reasonable to assume that those who kept their hearing aids past 90 days may be a self-selecting group with a slightly elevated baseline level of satisfaction with the devices. However, while device usage and experience has been shown to be linked to satisfaction (Uriarte et al., 2005; Vestergaard, 2006; Vestergaard Knudsen et al., 2010; Dashti et al., 2015), as the U.S. norm data also included responses from experienced hearing aid users (Cox and Alexander, 1999), we believe reasonable comparisons could still be made.

Another aspect to consider is that the SADL norms consisted of hearing aid satisfaction data from both private-pay and sponsored samples. While there was no difference observed between private-pay and sponsored respondents on global satisfaction, there was a difference in the SC subscale satisfaction score (Cox and Alexander, 1999). While we excluded the SC subscale items in both the retrospective and prospective cohort studies, we must acknowledge the inherent and implicit impacts of using a provisioned hearing aid on the expectations and perceptions of our prospective cohort participants. Our prospective cohort data were consistent with other studies where hearing aid cost was fully or partially sponsored demonstrating slightly elevated SADL scores compared to the published U.S. norms (Uriarte et al., 2005; Iwahashi et al., 2013; Dashti et al., 2015).

For the prospective cohort, the positive impact of self-fitting hearing aid use on QoL areas varied by area, but ranged from 19% of respondents endorsing improved physical health to 100% of respondents endorsing improved romantic life. Out of the 14 areas surveyed, all but three had unanimous responses of improvement or stability since using the investigational devices. In areas related to social functioning, 65.4% endorsed improvements in social life, 60% reported being better able to participate in group activities, and many reported improved relationships at home (64%) and at work (61.1%). Short-term hearing aid use was associated with an improved ability to communicate effectively in 69.2% of respondents. In areas related to sense of self, 60.9% reported improved feelings about oneself, 54.2% reported improved confidence, and 39.1% reported improved senses of safety and independence, respectively. Overall, improvements were endorsed by users across a number of QoL areas, particularly those related to communication, interpersonal relationships, and social functions. It is not entirely clear if and how the QoL responses may change with an extended duration of device wear. Future work may assess QoL improvements from a larger, real-world cohort. Our intent was to present these QoL data purely as descriptive findings; it would not be appropriate to directly compare our observations against those published in the MarkeTrak reports based on paying hearing aid customers. However, it was still interesting to note, with caution, that the endorsement of improvements observed with wearers of self-fitting OTC hearing-aid from this study were better than the responses reported with prescription hearing-aids fit by an audiologist following clinical best practice methods across several categories (Picou, 2022).

## 5 Conclusion

Taken together, and in considering some of the limitations of our study above, data involving new and experienced users of an investigational self-fitting OTC hearing aid suggest that users report a level of satisfaction and subjective benefit equivalent to or better than (in most areas assessed, with an exception on the PE subscale where the retrospective cohort reported lower satisfaction), those experienced by users of prescription hearing aids fit by audiologists following clinical best practice methods. Converging evidence from the retrospective and prospective cohorts with respect to consistent user-reported device usage and positive PI scores demonstrate the device category’s potential impact on hearing aid use adoption. With the establishment of the OTC category of hearing aids, there is hope that access to hearing healthcare will broaden. Given that untreated hearing loss has negative implications on many aspects of physical, cognitive, and emotional health, breaking down barriers to device access can ultimately have an outsized impact on objective and subjective outcomes; certainly, in our small sample of prospective self-fitting OTC hearing aid users, many endorsed experiencing improvements in relationships and other social situations. While more research is needed to fully characterize the potential positive impact that self-fitting OTC hearing aids may have on health outcomes, here we present preliminary but encouraging evidence that the use of self-fitting OTC hearing aids can play a role in helping to preserve or improve perceived quality of life for adults with mild to moderate hearing loss.

## Data availability statement

The raw data supporting the conclusions of this article will be made available by the authors, without undue reservation.

## Ethics statement

The studies involving humans were approved by Eargo Legal and Regulatory Review. The studies were conducted in accordance with the local legislation and institutional requirements. The participants provided their written informed consent to participate in this study.

## Author contributions

TS: Conceptualization, Data curation, Formal analysis, Investigation, Methodology, Project administration, Supervision, Validation, Writing – original draft, Writing – review & editing. LP: Formal analysis, Investigation, Writing – review & editing. JG: Methodology, Writing – original draft, Writing – review & editing. XC: Conceptualization, Data curation, Formal analysis, Investigation, Methodology, Project administration, Software, Validation, Writing – review & editing. JS: Conceptualization, Formal analysis, Methodology, Supervision, Writing – original draft, Writing – review & editing.
